# Application of the Gastrointestinal Simulator (GIS) Coupled with In Silico Modeling to Measure the Impact of Coca-Cola^®^ on the Luminal and Systemic Behavior of Loratadine (BCS Class 2b)

**DOI:** 10.3390/pharmaceutics12060566

**Published:** 2020-06-18

**Authors:** Bart Hens, Marival Bermejo, Rodrigo Cristofoletti, Gregory E. Amidon, Gordon L. Amidon

**Affiliations:** 1 Department of Pharmaceutical Sciences, College of Pharmacy, University of Michigan, Ann Arbor, MI 48109-1065, USA; bart.hens@kuleuven.be (B.H.); mbermejo@umh.es (M.B.); geamidon@umich.edu (G.E.A.); 2 Department of Pharmaceutical and Pharmacological Sciences, Faculty of Pharmaceutical Sciences, KU Leuven, Herestraat 49, 3000 Leuven, Belgium; 3 Department Engineering Pharmacy Section, Miguel Hernandez University, San Juan de Alicante, 03550 Alicante, Spain; 4 Center for Pharmacometrics and Systems Pharmacology, Department of Pharmaceutics, College of Pharmacy, University of Florida, Orlando, FL 32827, USA; rcristofoletti@cop.ufl.edu

**Keywords:** biopharmaceutics classification system (BCS), low aqueous solubility, oral bioavailability, oral drug absorption, in silico modeling, biopredictive dissolution testing, physiologically-based biopharmaceutics modeling (PBBM)

## Abstract

In the present work, we explored if Coca-Cola^®^ had a beneficial impact on the systemic outcome of the weakly basic drug loratadine (Wal-itin^®^, immediate-release formulation, 10 mg, generic drug product). To map the contribution of underlying physiological variables that may positively impact the intestinal absorption of loratadine, a multi-compartmental and dynamic dissolution device was built, namely the Gastrointestinal Simulator (GIS). The luminal behavior of one immediate-release (IR) tablet of 10 mg of loratadine was tested under four different fasted state test conditions in the GIS: (i) with 250 mL of water and applying a predetermined gastric half-life (t_1/2,G_) of 15 min; (ii) with 250 mL of water and applying a t_1/2,G_ of 30 min; (iii) with 250 mL of Coca-Cola^®^ and a t_1/2,G_ of 15 min; (iv) with 250 mL of Coca-Cola^®^ and a t_1/2,G_ of 30 min. After initiating the experiments, solution concentrations and solubility were measured in the withdrawn samples, and pH was monitored. To address the impact of the present CO_2_ in Coca-Cola^®^ on the disintegration time of the tablet, additional disintegration experiments were performed in a single-vessel applying tap water and sparkling water as dissolution media. These experiments demonstrated the faster disintegration of the tablet in the presence of sparkling water, as the present CO_2_ facilitates the release of the drug. The buffer capacity of Coca-Cola^®^ in the presence of FaSSGF was 4-fold higher than the buffer capacity of tap water in the presence of FaSSGF. After performing the in vitro experiments, the obtained results were used as input for a two-compartmental pharmacokinetic (PK) modeling approach to predict the systemic concentrations. These simulations pointed out that (i) the present CO_2_ in Coca-Cola^®^ is responsible for the enhancement in drug release and dissolution and that (ii) a delay in gastric emptying rate will sustain the supersaturated concentrations of loratadine in the intestinal regions of the GI tract, resulting in an enhanced driving force for intestinal absorption. Therefore, co-administration of loratadine with Coca-Cola^®^ will highly likely result in an increased systemic exposure compared to co-administration of loratadine with tap water. The mechanistic insights that were obtained from this work will serve as a scientific basis to evaluate the impact of Coca-Cola^®^ on the systemic exposure of weakly basic drugs for patients on acid-reducing agents in future work.

## 1. Introduction

During the late stages of oral drug product development, bioequivalence studies are performed to confirm the efficacy and safety of the tested product before marketing. Guidelines are provided by regulatory authorities; however, there is a need to harmonize these standardized protocols between different world regions. For instance, current U.S. Food & Drug Administration (FDA) bioequivalence guidelines promulgate co-administration of 240 mL of water to test the performance of the drug under fasted state conditions, whereas guidelines issued by the European Medicines Agency (EMA) indicate the administration of at least 150 mL of liquid to reflect fasting state conditions. In Japan, oral drug products are tested with 150 mL of water in clinical studies, according to the guideline of the Pharmaceuticals and Medical Devices Agency (PDMA) [1,2,3]. A recent survey study revealed that 895 adults (16 years and older), living in Flanders, Belgium, took their medication with a sip or half glass of water [4]. Concerning the type of beverage that was mostly consumed, water was the most preferred beverage (92.2%) followed by soda (12.9%), coffee (8.4%), juice (4.8%), milk (4.1%), and tea (2.7%). It should be noted that patients were able to indicate more than one specific beverage.

The intestinal absorption of drugs may be significantly altered as a wide variety of GI processes can be influenced by the type of co-administered beverage. For example, the concomitant intake of coffee or tea together with a drug product can cause an interaction at the level of metabolism as caffeine is both a substrate and inhibitor of CYP1A2 [5,6]. Served and ingested at a temperature of 50 °C, these hot beverages may alter the dynamics of the gastric emptying process as such [5,7]. For acidic carbonated beverages (i.e., sodas), a beneficial impact on the systemic concentrations of numerous weakly basic drugs has been observed [8]. To illustrate, an increase in plasma C_max_ and area under the curve (AUC) was observed for posaconazole after intake of the drug with Coca-Cola^®^ compared to water [9]. More recently, Hens and colleagues further explored which mechanisms are responsible for a higher systemic exposure of atazanavir after intake with a glass of Coca-Cola^®^ [10]. Five healthy subjects were asked to ingest one capsule of atazanavir (150 mg) with (i) a glass of water, (ii) a glass of Coca-Cola^®^, or (iii) with a glass of water under achlorhydric conditions (i.e., elevation of gastric pH caused by concomitant intake of a proton-pump inhibitor; PPI). After the intake of the drug product, GI fluids were aspirated and analyzed for drug content. In parallel, blood samples were collected and gastric motility was recorded using high-resolution manometry (HRM). Based on the obtained results, a slight increase in systemic exposure was observed after intake of atazanavir with Coca-Cola^®^ compared to intake with a glass of water (mean plasma C_max_ (standard deviation): 0.51 (0.38) versus 0.41 (0.40) µM, respectively). Intake of the medicine under achlorhydric conditions resulted in negligible plasma concentrations with a mean plasma C_max_ of 17 nM. After analyzing the aspirated gastric and intestinal fluids for atazanavir, similar gastric concentration-time profiles were noticed for the water and Coca-Cola^®^ condition. However, intestinal concentration-time profiles were different: supersaturated concentrations of atazanavir were more pronounced for a longer period after intake of the drug with a glass of Coca-Cola^®^ compared to the water condition where supersaturated concentrations of the drug were less pronounced and a higher extent of precipitation was observed. Based on the HRM recordings, it was observed that gastric contractions were delayed after intake of the drug with Coca-Cola^®^: the strong phase 2 contractions were absent for approximately 80 min on average after intake of the drug with Coca-Cola^®^, which is highly likely responsible for slowing down the gastric emptying process due to the present calories in the Coca-Cola^®^ (105 kcal for 250 mL of Coca-Cola^®^).

Although it would not be feasible to test drug products with Coca-Cola^®^ in the different clinical phases of drug development (due to potential addiction issues), it would be interesting to see if the systemic outcome of a drug compound can be positively affected by the concomitant intake of the drug with a glass of Coca-Cola^®^, a situation which would not be unusual to occur in daily life. Especially in the case of weakly basic compounds (e.g., ketoconazole, itraconazole, carbamazepine, erlotinib, atazanavir) [10,11,12,13,14], an increase in systemic exposure of the drug can be expected when gastric drug dissolution will be favored by the presence of Coca-Cola^®^ due to (i) its impact on the physiology (e.g., delay in gastric emptying, fluid volume changes) and/or due to (ii) the specific characteristics of the drink (pH 2.48, CO_2_) which are different compared to the characteristics of tap water (pH 7, no CO_2_). For numerous compounds, as listed above, this increase in oral bioavailability has been observed but there is no clarification with respect to what happens at the level of the GI tract that can explain the underlying mechanisms.

Therefore, this study aimed to investigate the potential beneficial impact of Coca-Cola^®^ on the luminal and systemic behavior of the weakly basic drug, loratadine. Loratadine is a weakly basic compound with a basic pKa of 5.3 and logP of 3.9 [15,16]. Based on previous work, loratadine showed the tendency to precipitate after inducing a pH-shift or formulating the drug as an oral lipid-based formulation [15,16]. Concerning its low solubility at the level of the small intestine, an enabling formulation strategy is a logical option to increase the bioavailability of loratadine. However, to the extent of our knowledge, there is currently no marketed enabling formulation of loratadine available. Therefore, it would be extremely interesting to explore if the oral bioavailability of loratadine may increase when co-administered with Coca-Cola^®^ and which underlying mechanisms are responsible for that. This work aimed to focus on specific parameters that may have a potential impact on the luminal concentrations as, for example, the gastric emptying rate, pH, buffer capacity, and sparging CO_2_ present in the drink. To do so, one single immediate-release (IR) tablet of 10 mg of loratadine (Wal-itin^®^, Walgreens, generic drug product, Ann Arbor, MI, USA) was tested under four different scenarios in the Gastrointestinal Simulator (GIS), varying the gastric emptying rate half-life (t_1/2,G_; 15 min versus 30 min) and the co-administered beverage (tap water versus Coca-Cola^®^). After initiating the experiments, solution concentrations and solubility were measured in the withdrawn samples. Buffer capacity of the initial gastric media was tested in the presence and absence of CO_2_. To address the impact of CO_2_ on the release of the drug, additional in vitro disintegration experiments were performed in single-vessel units where the disintegration of the tablet was visually checked in the presence of tap water or sparkling water.

After performing these experiments, the obtained dissolution data were used as input for a two-compartmental pharmacokinetic (PK) model to describe the absorption, distribution, and clearance of the drug. These four test conditions illustrate the possible scenarios that may occur in vivo, by changing one parameter at a time (i.e., co-administered beverage and/or the gastric emptying rate). Based on these outcomes, the impact of these covariates can be quantified and predictions can be made capturing the systemic outcome of the drug when taken with a glass of Coca-Cola^®^.

## 2. Materials and Methods

### 2.1. Chemicals

Loratadine (Wal-itin^®^) was the drug compound of interest and purchased from Walgreens (Ann Arbor, MI, USA). Wal-itin^®^ is a generic, immediate-release (IR) formulation containing 10 mg of loratadine. Acetonitrile was obtained from VWR International (West Chester, PA, USA). Methanol, HCl and trifluoroacetic acid (TFA) were purchased from Fisher Scientific (Pittsburgh, PA, USA). NaOH, NaCl and NaH_2_PO_4_·H_2_0 were received from Sigma-Aldrich (St. Louis, MO, USA). Purified water (filtrated and deionized) was used for the analysis methods and dissolution studies to prepare the dissolution media (Millipore, Billerica, MA, USA). Simulated intestinal and gastric fluid (SIF/SGF) powder was purchased from Biorelevant.com Ltd. (London, UK), and the media were prepared by the supplier’s protocol. Coca-Cola^®^ and Chaudfontaine^®^ sparkling water were provided by The Coca-Cola Company (Atlanta, GA, USA). The co-administered beverage to simulate fasting state conditions was tap water.

### 2.2. Representing the Physicochemical Properties of Loratadine by Using the ADMET Predictor

To calculate the physicochemical properties of loratadine, the ADMET Predictor version 9.5 was applied (Simulation Plus^™^, Lancaster, CA, USA). The ADMET Predictor was used to describe both the physicochemical properties (e.g., basic pKa, LogP, molecular weight) and the metabolic pathways (e.g., interaction of metabolic enzymes and formed metabolites). ADMET Predictors use appropriate algorithms that allow the use of theoretical models to reflect the behavior of the drug in the human body, allowing in a short time to estimate the possibility of toxic effects, as well as assessing the ADMET parameters of the molecule based on the chemical formula. Each of the determined parameters has appropriate ranges of values based on which it is possible to estimate the behavior of the drug in the human body.

### 2.3. Design of the In Vitro Dissolution Studies Performed with the Gastrointestinal Simulator (GIS)

**Figure 1:**
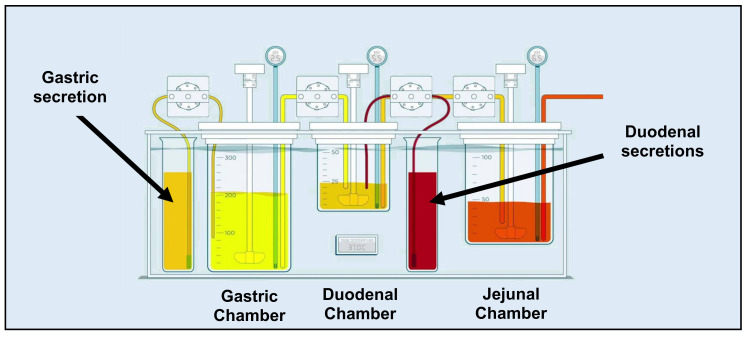
Setup and design of the GIS that was applied to test Wal-itin^®^ (immediate-release generic drug formulation, 10 mg of loratadine) in fasted state conditions under different test conditions.

One of the models that thoroughly explored the potential of supersaturation for weakly basic drugs and how these compounds start to precipitate after GI transfer is the Gastrointestinal Simulator (GIS) [17]. This multi-compartmental device exists of three different chambers (i.e., a gastric chamber (GIS_Stomach_), a duodenal chamber (GIS_Duodenum_), and a jejunal chamber (GIS_Jejunum_)), reflecting the human stomach, duodenum, and jejunum and also reflecting the physiological conditions in terms of gastric emptying rate, secretions, and residual pH. The design of the GIS is depicted in Figure 1.

Four different test conditions were proposed to mechanistically explore the luminal behavior of loratadine and to measure the impact of Coca-Cola^®^ on luminal behavior:

1. With 250 mL of water and a predetermined t_1/2,G_ of 15 min **(Condition 1)**;
2. With 250 mL of water and a predetermined t_1/2,G_ of 30 min **(Condition 2)**;
3. With 250 mL of Coca-Cola^®^ and a predetermined t_1/2,G_ of 15 min **(Condition 3)**;
4. With 250 mL of Coca-Cola^®^ and a predetermined gastric t_1/2,G_ of 30 min **(Condition 4).**

A specific focus went out to the (i) co-administered beverage (water versus Coca-Cola^®^) and (ii) the rate of gastric emptying. The caloric content of Coca-Cola^®^ (105 kcal for 250 mL) is responsible for delaying gastric emptying [18] and, therefore, two different gastric emptying rates were tested: a 15 min t_1/2,G_ (reflecting gastric emptying in fasted state conditions) and a 30 min t_1/2,G_ (reflecting a two-fold delay in gastric emptying caused by the intake of Coca-Cola^®^). These selected t_1/2,G_ are in line with the measured t_1/2,G_ as measured in humans under fasted (estimated t_1/2,G_between 4 and 13 min) and fed state conditions (estimated t_1/2,G_ between 21 and 40 min). The dissolution media, initial volumes, and secretion rates are described in Table 1.

**Figure 2:**
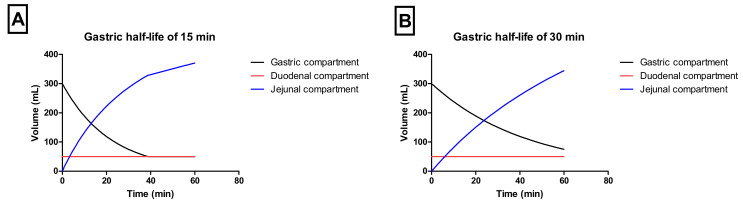
The representative volumes throughout the different chambers of the GIS when applying a t_1/2,G_ of (**A**) 15 min or (**B**) 30 min.

Gastric emptying occurred by a first-order kinetic process with a t_1/2,G_ of 15 or 30 min. Duodenal volumes were kept constant during the entire experiment (50 mL). The jejunal compartment was left empty initially (i.e., no volume present) (Figure 2A,B).

As soon as the experiment started, the gastric content was immediately transferred to the GIS_Duodenum_ by applying a transfer tube and a peristaltic pump (Ismatec REGLO pump; IDEX Health and Science, Glattbrugg, Switzerland). Constant gastric and duodenal secretions (1 mL/min) were ongoing during the entire experiment, regulated by two peristaltic pumps. To assure that the duodenal volume remained constant during the entire experiment, the in- and outflow transfer rates were equal to keep the duodenal volume at a constant volume of 50 mL. All transfer pumps were calibrated before the start of the experiments using purified water. To mimic hydrodynamics in the gastric and duodenal chambers, the CM-1 overhead paddles (Muscle Corp., Osaka, Japan) stirred with a specific rate of 20 rotations per minute (rpm). These rotations were interspersed with quick burst every 25 s to mimic the more stronger phase 2 and 3 contractions of stomach and duodenum which occur during the MMC cycle; the weaker distal contractions of the intestinal tract were simulated in the GIS_Jejunum_ by stirring at a constant rate. No bursts were introduced in this chamber. Two pH electrodes (Thermo Scientific, Orion 525A+, Waltham, MA, USA) were located in the GIS_Stomach_ and GIS_Duodenum_ to record the pH during the duration of the experiment. Only during one out of three experiments was pH monitored. Before using these pH electrodes, calibration was performed at pH 1, 4, and 7 to ensure accurate and precise pH measurements. All experiments were performed at 37 °C. At predetermined time points, samples were withdrawn from the GIS compartments up to 60 min to measure (i) the dissolved amount of loratadine and the (ii) thermodynamic solubility of loratadine. The pH electrodes, pumps, and overhead paddles were controlled by a ‘do-it-yourself’ in-house computer software program. Solution concentrations were determined by centrifuging 200 µL of the withdrawn sample for 5 min at a gravitational acceleration of 17,000 g (AccuSpin Micro 17, Fisher Scientific, Pittsburgh, PA, USA). After centrifugation, the supernatant was directly five-fold diluted with methanol to capture the dissolved fraction. Finally, the thermodynamic solubility of loratadine was determined by the shake-flask method, incubating the withdrawn samples for 24 h with an excess amount of loratadine. All obtained samples were analyzed by HPLC (see below). All experiments were performed in triplicate except for pH measurements (*n* = 1).

### 2.4. Analysis of the Mass Transport of Loratadine throughout the GIS

A mass transport analysis (MTA) was developed to describe the passage of dissolved loratadine throughout all the chambers of the GIS. Mass transport equations for the GIS were constructed based on the drug dissolution, precipitation, and transit kinetics as earlier described for dipyridamole and posaconazole by Matsui and co-workers and Bermejo and colleagues, respectively [19,20]. All these equations were adopted to describe the mass transport of loratadine, with slight modifications specific to the physicochemical parameters of loratadine (Table 2). Mathematical equations are included in the supplementary materials (S1).

During all experiments, the administered dose was 10 mg of loratadine. Secretion rates in the gastric and duodenal chamber are given by k_sec_s_ and k_sec_d_, respectively; t_1/2,G_ represents the gastric emptying half-life; V_s_, V_d_, and V_j_ represent the gastric, duodenal and jejunal volumes. Z_S, Z_D, and Z_J are dissolution coefficients, considering the pH-dependent dissolution and solubility of loratadine. The term ‘Frac’ is referring to the fraction of particles that are transferred throughout the different compartments. Consequently, ‘1-Frac’ refers to the fraction of particles that were not transferred throughout the different chambers of the dissolution device. Precipitation rates are described as first-order kinetic processes listed by k_pre_d_ and k_pre_j_ for ongoing precipitation in the duodenal and jejunal chamber, respectively.

### 2.5. Measuring of the Buffer Capacity

The buffer capacity of (i) 50 mL of FaSSGF (pH 2) + 250 mL of Coca-Cola^®^, (ii) 50 mL of FaSSGF (pH 2) + 250 mL of tap water, (iii) 50 mL of FaSSGF (pH 2) + 250 mL of degassed Coca-Cola^®^, and (iv) 50 mL of FaSSGF (pH 2) + 250 mL of sparkling water was measured. Coca-Cola^®^ was degassed by stirring for two hours at 37 °C. Before the start of the experiment, a quick burst of stirring was performed to ensure that residual CO_2_ bubbles were removed from the beverage. The first two scenarios represent the initial starting conditions in the gastric chamber. The final two conditions were performed to measure the impact of CO_2_ on the buffer capacity of the gastric media. Buffer capacity (*β*) is the ability of the buffer to keep the pH stable and can be calculated as follows:

(1) $$\beta =\frac{\Delta n}{\Delta pH}$$

where ∆*n* stands for the equivalence of strong acid or base added per volume liter and ∆*pH* is the change in pH. The buffer capacity of Coca-Cola^®^ is tested by measuring the volume of NaOH (1 M) and HCl (1 M) needed to alter the pH with 1 unit. The pH electrode was calibrated at pH 1, 4, and 7 to ensure accurate pH measurements. The buffer capacity was calculated using Equation (1). Data are presented as mean + SD (*n* = 3).

### 2.6. Disintegration Experiments to Assess the Impact of CO_2_ on the Release of the Drug

To address the impact of CO_2_ on the release of loratadine, disintegration studies were performed in 250 mL of tap water (i.e., in absence of CO_2_) and 250 mL of sparkling water (i.e., in the presence of CO_2_; with a concentration of bicarbonate of 305 mg/L and a pH of 4.94) (Chaudfontaine^®^ red label; The Coca-Cola Company, Atlanta, GA, USA). Disintegration experiments were performed at 37 °C. The dissolution beakers contained a magnetic stirrer to induce hydrodynamics (70 rpm). An iron coil was used to keep the tablet at a certain level in the dissolution beaker, assuring no direct contact with the magnetic stirrer on the bottom of the vessel. After 30 min, the formulations were removed from the vessel and an evaluation was made concerning the remaining size of the tablet. Experiments were performed in triplicate.

### 2.7. In Silico Simulations to Predict the Systemic Pharmacokinetic (PK) Profiles of Loratadine

**Figure 3:**
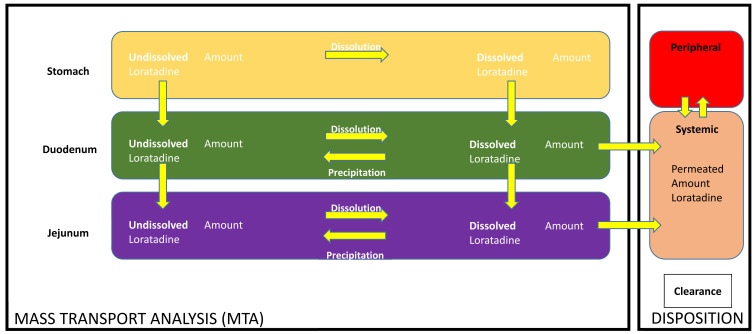
Mass transport analysis model (MTA) to simulate dissolution, precipitation, and transit of loratadine as observed in the GIS when testing the different test conditions coupled with in silico disposition parameters to simulate plasma profiles of loratadine for all four test conditions. ‘CL’ means ‘clearance of the drug’.

A two-compartmental open PK model was developed to predict the plasma profiles after administration of loratadine under the four test conditions. Fitting and simulations were performed with Phoenix WinNonlin^®^ version 7.0 (Certara, Princeton, NJ, USA). A second PK analysis was performed using PK Solver 2.0 (Microsoft Excel^®^, Redmond, WA, USA), demonstrating similar results to the Phoenix WinNonlin^®^ analysis kit (data not shown). Twelve independent PK studies were used to develop the compartmental PK model and the PK parameters are summarized in Table 3. Integrated MTA and disposition models, previously described by Matsui et al., were adjusted to loratadine (Figure 3) [19].

### 2.8. Analysis of Loratadine by HPLC

All samples derived from the GIS studies were analyzed for loratadine by HPLC-UV (Hewlett Packard series 1100 HPLC Pump combined with Agilent Technologies 1200 Series Autosampler (Santa Clara, CA, USA). A volume of 100 µL was injected into the HPLC system connected to a UV-lamp that was able to detect loratadine at a wavelength of 248 nm (Agilent 1100 Series UV lamp). An isocratic run with a mixture of 70% acetonitrile and 30% purified water was used to detect loratadine using a C-18 column (Kinetex C18 HPLC column, 250 × 4.60 mm^‒5^ micron, Phenomenex, Golden, CO, USA) and a 1 mL/min flow rate. Calibration curves were made in methanol, based on a stock solution of loratadine in methanol (0.1 mg/mL). Linearity was observed between 40 µg/mL and 0.156 µg/mL with a regression coefficient of at least 0.995 between the AUC of the obtained peaks versus the spiked concentrations. The peaks were integrated using ChemStation software (Agilent Technologies, B.04.03 version, Santa Clara, CA, USA).

### 2.9. Data Analysis and Presentation

Dissolution profiles of loratadine derived from the GIS were plotted as a function of time and were also expressed as the observed degree of supersaturation (DS). The DS was expressed as:

(2) $$DS=\frac{C}{C_{eq}}$$

where C is the dissolved concentration of loratadine at a specific time point and $C_{eq}$ is the thermodynamic solubility of loratadine at that same time point. Solution concentrations of loratadine were plotted as a function of time throughout the different chambers of the GIS and presented as mean + SD (*n* = 3). All data were plotted using Graphpad Prism 5.00 for Windows 10 (GraphPad Software, San Diego, CA, USA). Only descriptive statistics were performed to analyze the data.

## 3. Results and Discussion

### 3.1. Physicochemical Characteristics of Loratadine

**Figure 4:**
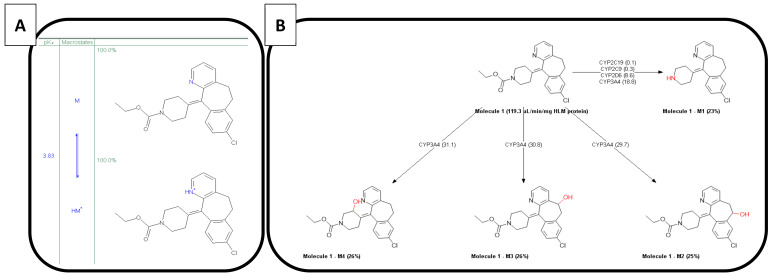
(**A**) the ionized versus unionized distribution of loratadine and (**B**) the metabolic pathways of loratadine. These predictions were performed in the ADMET Predictor version 9.5 of Simulations Plus^™^.

Loratadine is categorized as a Biopharmaceutics Classification System 2b compound (BCS class 2b; weak base) with an estimated basic pKa of 3.83 and a predicted cLogP of 4.54 using the ADMET Predictor 9.5 (Simulations Plus™, Lancaster, CA, USA). The molecular weight of loratadine is 382.89 g/mol. As mentioned by other authors, this estimated pKa and LogP value differs from the one stated by Stillhart et al. (basic pKa of 5.3 and LogP value of 3.9) [15]. The molecule has one major active metabolite (descarbethoxyloratadine), being four times more active than the parent drug and showing a longer elimination half-life (20 versus 10 h, respectively) [33,34]. The ionized versus unionized microspecies distribution and the metabolic pathways are depicted in Figure 4A,B, respectively. Due to the ionization of the nitrogen present in the pyridine group, the compound becomes more soluble in the acidic environment of the stomach, which will lead to increased gastric concentrations of the drug. After GI transfer, a drop in solubility will occur due to the neutral pH environment that will convert the ionized form to the unionized/ neutral form, which will induce precipitation of the drug in the intestinal tract.

### 3.2. Dissolution Kinetics of Loratadine under Different Dosing Conditions in the GIS: A Mechanistic Approach

**Figure 5:**
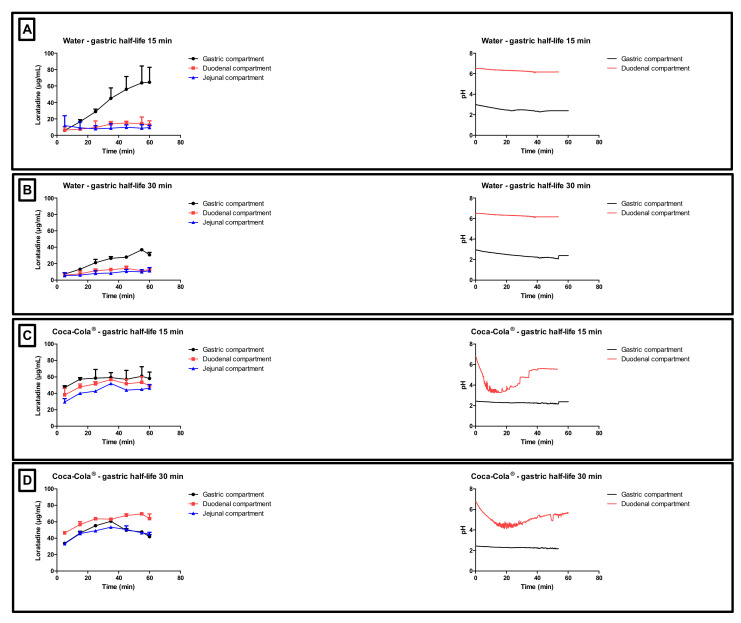
Gastric, duodenal and jejunal concentration-time profiles of loratadine obtained after performing (**A**) condition 1, (**B**) condition 2, (**C**) conditions 3, and (**D**) condition 4 in the Gastrointestinal Simulator (GIS). Data are presented as mean + SD (*n* = 3). The right side represents the corresponding gastric and duodenal pH values as a function of time. pH values were measured during one set of experiments (*n* = 1).

In addition to the GI pH, one may concern the gastric residence time as another factor that could impact the systemic exposure of weak bases. Four different conditions were tested in the GIS and the respective concentration-time profiles are depicted in Figure 5. The inserts show the measured pH values as a function of time throughout the gastric and duodenal chambers of the GIS.

With respect to conditions 1 and 2 (water), high gastric concentrations were observed due to the acidic content of the chamber which stimulates the dissolution of the weak base. Gastric concentrations were lower for condition 2 compared to condition 1 since there is a two-fold slower rate of gastric emptying, resulting in more volume available for dissolution, which results, on its turn, in lower gastric concentrations (ratio of amount over volume). Nevertheless, the dissolved amount of loratadine between both test conditions is similar. Concerning the duodenal and jejunal concentrations, a rapid decrease was observed due to (i) a dilution effect when being transferred from the gastric compartment to the intestinal compartments and (ii) intestinal precipitation. Co-administration of loratadine with Coca-Cola^®^, regardless of the applied gastric emptying time, resulted in a faster release and dissolution of the drug in the gastric chamber compared to the water conditions (i.e., conditions 1 and 2 versus 3 and 4).

**Figure 6:**
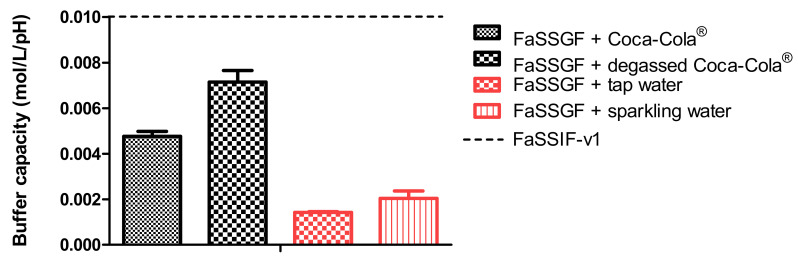
Buffer capacity of fasted state simulated gastric fluid (FaSSGF) in combination with (**i**) Coca-Cola^®^, (**ii**) degassed Coca-Cola^®^, (**iii**) tap water and (**iv**) sparkling water. The dotted line represents the buffer capacity of fasted state simulated intestinal fluids version 1 (FaSSIF-v1). Data presented as mean + SD (*n* = 3).

In the experiments with Coca-Cola^®^, the acidic load to the GIS duodenal chamber resulted in an immediate pH drop. However, this drop was more pronounced for condition 3 than for condition 4 as the rate of gastric emptying was twice as fast for condition 3. The minimal pH that was observed in the duodenal chamber was pH 3.27 and pH 4.25 for conditions 3 and 4, respectively. The reason why the drop for conditions 3 and 4 was more pronounced compared to conditions 1 and 2 is related to the buffer capacity of the gastric content: a 4-fold higher buffer capacity of FaSSGF + Coca-Cola^®^ (Figure 6) outweighed the neutralizing capacity of the duodenal content (i.e., residual volume and secretions).

It is debatable if such a drop in duodenal pH, as observed for condition 3, will be observed after the administration of Coca-Cola^®^ to healthy subjects. Published values by Hens et al. and Walravens et al. showed on average no significant differences between duodenal pH values after dosing the drug with tap water or with Coca-Cola^®^. Although the buffer capacity of the gastric content may be enhanced when drinking Coca-Cola^®^, the delayed transfer from the stomach to the small intestine may control the intestinal pH at a certain value equal as observed for fasted state conditions. The buffer capacity of FaSSIF-v1 is equal to 0.010 mol/L/∆pH, which is still higher than the buffer capacity values as observed for the media present in the gastric chamber of the GIS [35]. Crucial to note is that, although the initial gastric pH is similar among all test conditions, fluctuations in duodenal pH were only observed for conditions 3 and 4, which show the importance of also taking into account buffer capacity and not only gastric pH. Polster et al. investigated the impact of Sprite^®^ (pH 3.3) on the systemic exposure of the weak base Lilly Compound X (LCX). The authors simulated two different gastric pH conditions (pH 2 versus 4.5) to investigate the impact on duodenal concentrations in the artificial stomach-duodenum (ASD) model. The initial gastric conditions demonstrated having a significant impact on the outcome of the drug [36]. Fluctuations in intestinal pH are not uncommon as recently observed in the aspirated fluids of healthy subjects [37]. By applying physiologically-based pharmacokinetic (PBPK) modeling, Fotaki and Klein concluded that a possible increase in systemic exposure of itraconazole is not necessarily related to a dynamic change in GI pH but also caused by other variables (such as, for instance, prolonged gastric residence time) [38]. When setting a delay in gastric emptying time as done for the experiments during condition 4, measured duodenal pH values were in line with the published pH values from Hens et al. and Walravens et al. [9,10] To measure the impact of the present CO_2_ on the buffer capacity of the dissolution media, the buffer capacity of degassed Coca-Cola^®^ and sparkling water was measured. No substantial impact of the present CO_2_ on the buffer capacity was shown. Moreover, no significant difference was observed in pH between degassed Coca-Cola^®^ and Coca-Cola^®^ as such (pH 2.48).

**Figure 7:**
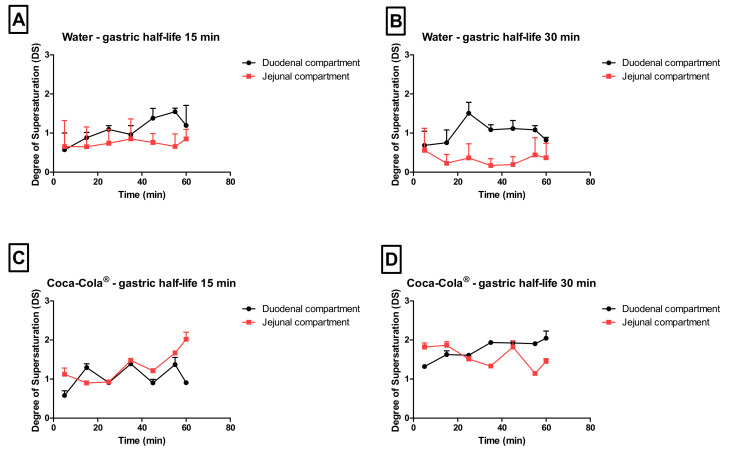
The observed degree of supersaturation (DS) as a function of time in the duodenal and jejunal compartments for (**A**) condition 1, (**B**) condition 2, (**C**) condition 3, and (**D**) condition 4. Data presented as mean + SD (*n* = 3).

As the gastric dissolution of the drug occurred very rapidly (highly likely due to the sparkling CO_2_), a high dissolved fraction of loratadine was immediately observed in the duodenal compartment, after GI transfer. With respect to the degree of supersaturation (DS), the maximal DS was higher for conditions 3 and 4 (Coca-Cola^®^) compared to conditions 1 and 2 (water), especially in the jejunal chamber of the GIS (Figure 7).

Besides the rapid gastric release, increased solution concentrations were observed for condition 4 when the rate of emptying was delayed from 15 to 30 min, indicating the importance of gastric emptying on (i) the obtained supersaturated concentrations in the intestinal segments and (ii) the duration of this metastable state.

The impact of different gastric emptying rates on precipitation kinetics of poorly soluble weak bases has thoroughly been investigated in vitro by Kostewicz and co-workers [39]. Authors concluded that a fast gastric emptying rate tends to lead to a higher supersaturated state of the drug compound but also a higher extent of precipitation. It was hypothesized that in vivo drug precipitation will be more pronounced when the drug is quickly delivered from the stomach into the intestine. Also in this case, when comparing conditions 3 and 4, a positive effect of the delay in gastric emptying on the intestinal concentrations can be observed. When increasing the ingested amounts of calories (> 105 kcal), this delay in gastric emptying can even be more pronounced [18,40].

In this experimental work, no distinction was made between duodenal secretions nor between different fluid volumes among the different test conditions as only one parameter was changed at a time (i.e., gastric emptying rate and/or co-administered beverage) to address the difference in outcome towards the one parameter that had changed. However, the in vivo situation might be more complex as the intake of a caloric beverage will alter the physiology according to ‘fed state-like’ conditions (e.g., delay in gastric emptying, stimulation of GI secretions) [18,41].

### 3.3. Disintegration of the Tablet in the Presence and Absence of CO_2_: Sparkling versus Still Water

**Figure 8:**
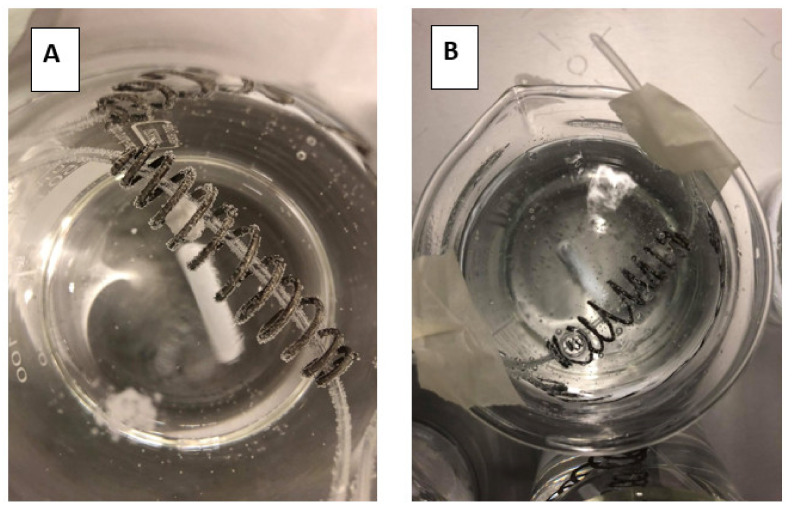
Top view of the dissolution beakers containing the loratadine tablet in the iron coil. (**A**) represents the disintegration test in the presence of still/tap water while (**B**) represents the disintegration test in the presence of sparkling water.

To measure the impact of the present CO_2_ on the release of the drug, a disintegration test was performed where the tablet remained for 30 min in 250 mL of tap water or sparkling water (Figure 8A,B, respectively).

**Figure 9:**
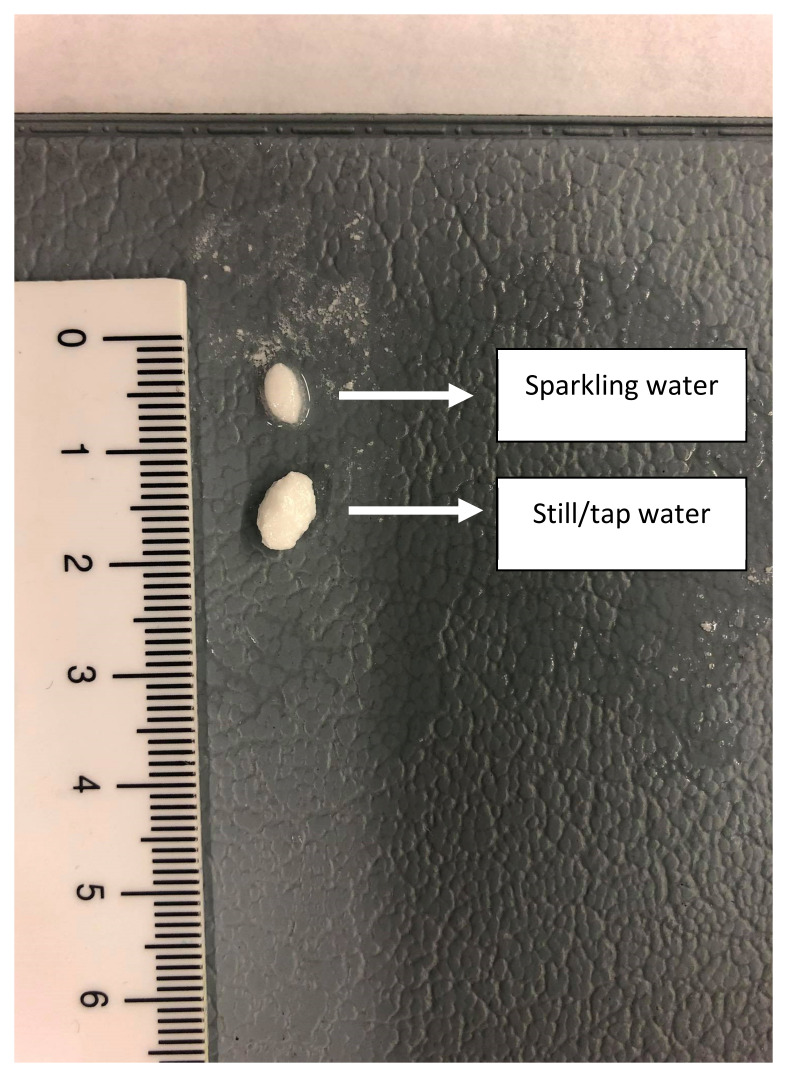
Two tablets representing the remaining tablet size after a 30 min disintegration test in sparkling and tap water.

After 30 min, the tablets were evaluated concerning their size as can be depicted in Figure 9.

Due to this fast release (rapid disintegration), loratadine will be quickly released and will easily dissolve in the gastric media of the stomach compartment, explaining the faster onset in dissolution kinetics for the Coca-Cola^®^ condition compared to the tap water condition. Carbon dioxide (CO_2_) has been shown to trigger the release of a solid dosage form, as already observed for acetaminophen when the drug was administered with a glass of sparkling water (direct effect) [42]. The authors observed transient pressure gradients in the distal part of the stomach which were not observed when the drug was administered with still water (indirect effect) [42]. An in vitro dissolution test in the USP 2 apparatus confirmed the beneficial impact of CO_2_ on the release of acetaminophen, regardless of the hydrodynamics induced by the stirring paddle (30 and 75 rpm). As gastric pH is comparable between all test conditions, it is more likely to accept that the present CO_2_ in Coca-Cola^®^ is the responsible factor for the enhanced release rather than the gastric pH. The same observations were made by Kelly and co-workers where researchers explored the disintegration time for a new formulation of paracetamol (Panadol Actifast^®^) containing 630 mg of sodium bicarbonate. The disintegration time for this formulation was compared to the conventional immediate-release formulation (Panadol^®^). The increase in dissolution rate is highly likely a result of turbulence caused by gaseous CO_2_ release at the level of the tablet/ dissolution fluid interface, leading to a disruption of the boundary diffusion layer. This was consistent with their proposed hypothesis that the generation of CO_2_, resulting from the reaction of sodium bicarbonate with HCl in the stomach, increases the rate of paracetamol dissolution from Panadol Actifast^®^ tablets compared to the conventional Panadol^®^ tablets due to CO_2_ release that will stimulate the disintegration of the tablet [43,44].

### 3.4. Mass Transport Analysis and In Silico Predictions of the Disposition of Loratadine

**Figure 10:**
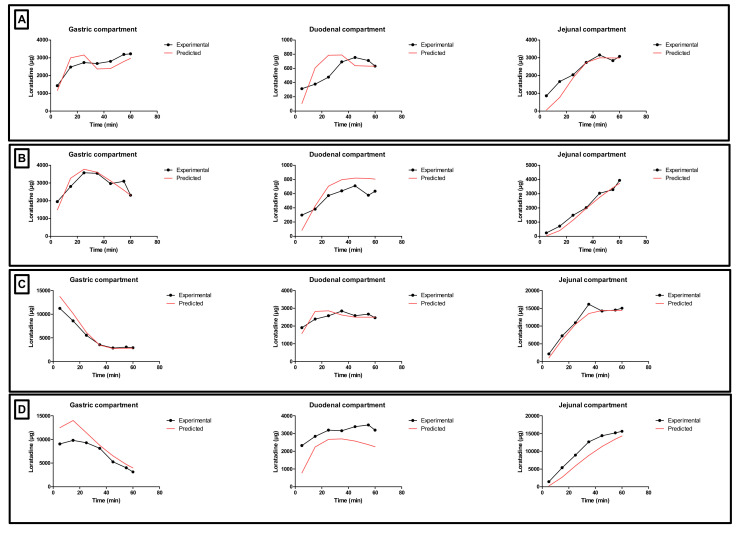
Gastric, duodenal, and jejunal concentration-time profiles of loratadine obtained after performing (**A**) condition 1, (**B**) condition 2, (**C**) condition 3, and (**D**) condition 4 in the GIS (black line) and by simulation (red line).

Since the GIS is a computer-controlled in vitro system of different connected compartments representing the different organs of the GI tract, a mass transport analysis model was developed to simulate the passage of the drug throughout these different chambers in terms of drug mass. Figure 10 shows the time-dependent mass transport of loratadine as measured in the GIS and the full lines represent the simulated profiles obtained by the mathematical equations representing the mass transport of loratadine throughout the different chambers of the GIS model.

**Figure 11:**
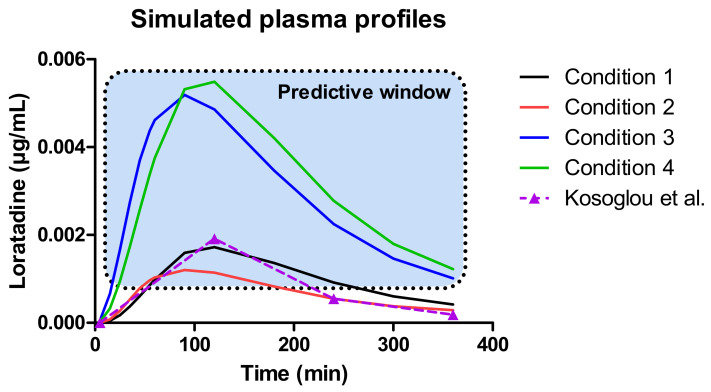
Simulated plasma concentration-time profiles of loratadine for the four different test conditions. To compare and to validate the fasted state results, the average systemic concentration-time profile of loratadine was depicted when one tablet of loratadine (10 mg, CLARITIN-D^®^ 24 h, RLD) was given orally to 24 healthy men under fasted state conditions [32].

Applying the mathematical equations as initially described by Matsui and co-workers, the predicted mass transport curves were in line with the measured mass transport curves derived from the GIS [19]. Dissolution coefficients were used to simulate the dissolved fraction of loratadine, taking into account the measured pH during the in vitro experiments as shown in Figure 5. In addition, the dynamic flow of volumes throughout the different chambers was considered when applying different speed rates for the peristaltic pumps to simulate the fast and slow gastric emptying process. To simulate the precipitation of loratadine in the intestinal chambers, precipitation rate constants were applied which were fitted to the experimental data. Applying these rate constants, an initial decrease in dissolved amount of loratadine was observed in the duodenal and jejunal compartments, representing the precipitation kinetics due to the pH-shift. Interestingly, to fit the curve, the duodenal precipitation rate constants were set at lower values for the conditions when a delayed gastric emptying rate was applied (conditions 2 and 4), which supports the hypothesis that a delayed gastric emptying will result in delayed precipitation kinetics (Table 2). It is worth mentioning that the formulation did not completely disintegrate during the 60 min dissolution experiment, and, therefore, this was taken into account during the modeling and simulation exercise. After obtaining these predicted values, a two-compartmental PK model was developed to describe the absorption, distribution, and clearance of loratadine for the different test conditions. The simulated plasma profiles are depicted in Figure 11.

The outcome of all predictions demonstrates a ‘predictive window’ that defines a certain space where the observed plasma concentrations can be situated when loratadine is co-administered with a glass of Coca-Cola^®^. As a reference, the average systemic concentration-time profile of loratadine after oral administration of a 10 mg dose (Claritin-D^®^ 24 h, RLD, Schering-Plough Corporation, Kenilworth, NJ, USA) under fasting conditions to healthy men was added to this figure, to demonstrate the accuracy and precision of our in silico simulations for the fasted state condition. Moreover, as the reference data are referring to the reference listed drug product (CLARITIN-D^®^), bioequivalence was shown for the Wal-itin^®^ formulation that was used during these dissolution experiments, when comparing the simulated plasma AUC_5-360 min_ (0.35 µg/mL.min) and C_max_ (0.0017 µg/mL) of Wal-itin^®^ for condition 1 versus the observed plasma AUC_5-360 min_ (0.30 µg/mL.min) and C_max_ (0.0019 µg/mL) of CLARITIN-D^®^. For both disposition parameters, less than 20% difference was observed between both formulation products, demonstrating bioequivalence for both drug products (reference versus generic).

A minimal difference in systemic exposure between conditions 1 and 2 was observed, showing the impact of a slower gastric emptying on the systemic outcome of loratadine. Gastric emptying can be classified as a possible co-variate explaining intersubject variability in PK studies. As condition 4 is the closest to the situation that would presumably occur in vivo, a three-fold increase in plasma C_max_ and AUC can be expected (best-case scenario). Nevertheless, as discussed before, the in vivo situation might be more complex as the intake of a caloric beverage will alter the physiology according to ‘fed state-like’ properties (e.g., change in residual fluid volumes). However, based on the observed results from other clinical studies where weak bases were administered with Coca-Cola^®^, it is suitable to accept that the delay in gastric emptying rate and the increased gastric release and dissolution are the two most responsible factors for the increase in systemic exposure of loratadine rather than other variables (e.g., enhanced secretions and fluid volumes).

## 4. Conclusions and Future Directives

In this work, in vitro dissolution experiments in the presence of Coca-Cola^®^ were conducted to evaluate the luminal disposition of the weakly basic drug, loratadine. Subsequently, the obtained in vitro dissolution data were used as an input for an in silico two-compartmental PK model to simulate plasma-concentration time profiles. Based on these results, it can be concluded that, due to (i) the stimulatory effect of CO_2_ on drug release and (ii) the delay in gastric emptying (caused by the caloric content of Coca-Cola^®^), a beneficial impact on luminal behavior can be accomplished, resulting in higher systemic concentrations than when loratadine would have been administered with a glass of water. In these four different test conditions, we specifically focused on: (i) the difference in the co-administered drink (i.e., water versus Coca-Cola^®^) and (ii) the modification of the gastric emptying rate due to the present calories in Coca-Cola^®^. Based on the simulated plasma concentration-time profiles for the four different test conditions, the GIS model was validated when comparing the water condition with clinical data from the literature where loratadine was administered under fasted state conditions. With respect to the Coca-Cola^®^ conditions, a three-fold increase in systemic exposure can be expected (best-case scenario). Based on other, independent studies where weakly basic drugs were co-administered with Coca-Cola^®^, it is more likely to accept that the delay in gastric emptying and enhanced drug release (triggered by the present CO_2_) will play a pivotal role to explain the increase in systemic concentrations. Over the years, the GIS model has been optimized and validated and is currently setting specific standards to test new or generic drug formulations by a simple approach: (i) exploring the luminal behavior of the drug product in the GIS, (ii) describing the mass transport in computational software, and (iii) coupling the mass transport with in silico modeling to reflect the distribution and clearance of the drug. By doing so, an interesting alternative has been launched that can hopefully replace in vivo studies to a certain extent. Future studies will focus on performing clinical studies where the impact of Coca-Cola^®^ on plasma C_max_ and AUC will be investigated in human subjects compared to fasting state conditions. In addition, the impact of Coca-Cola^®^ on plasma AUC and C_max_ will be investigated in humans who are on acid-reducing agents to explore how Coca-Cola^®^—until a certain level—may increase the oral bioavailability of weakly basic drugs under these circumstances.

## Figures and Tables

**Table 1:** Experimental conditions in each compartment for testing the different drug formulations of posaconazole in the GIS. The GIS_Jejunum_ was initially empty and no secretions were simulated in this chamber.

Experimental Test Conditions	GIS_Stomach_	GIS_Duodenum_	GIS_Jejunum_
**Dissolution Media**	Fasted state simulated gastric fluid (FaSSGF), pH 2	Fasted state simulated intestinal fluid version 1 (FaSSIF-v1), pH 6.5	/
**Initial Volume**	50 mL FaSSGF (pH 2) + 250 mL of tap water (**Conditions 1 and 2**) 250 mL of Coca-Cola^®^ (**Conditions 3 and 4**)	50 mL FaSSIF-v1	/
**Secretions**	1 mL/min of FaSSGF, pH 2	1 mL/min of FaSSIF-v1, pH 6.5 (4 x concentrated)	/

**Table 2:** Reference listed data as input for describing the dissolution, precipitation, and transit kinetics of loratadine for the different test conditions in the GIS. ‘t_1/2, G_’ means the gastric half-life.

	Condition 1	Condition 2	Condition 3	Condition 4	Reference
Dose (mg)	10	10	10	10	Generic Drug Product Wal-itin^®^ (Walgreens, Ann Arbor, MI, USA)
k_sec_s_ (mL/min)	1	1	1	1	[19]
k_sec_d_ (mL/min)	1	1	1	1	[19]
t_1/2,G_ (min)	15	30	15	30	[19]
V_s_ (mL)	300 to 5	300 to 5	300 to 5	300 to 5	[19]
V_d_ (mL)	50	50	50	50	[19]
V_j_ (mL)	0 to 390	0 to 390	0 to 390	0 to 390	[19]
Z_S (mL/µg/min)	1.43 × 10^−11^	1.72 × 10^−11^	1.51 × 10^−10^	7.41 × 10^−11^	Optimized by fitting
Z_D (mL/µg/min)	3.63 × 10^−11^	3.63 × 10^−11^	3.63 × 10^−11^	5.31 × 10^−11^	Optimized by fitting
Z_J (mL/µg/min)	5.05 × 10^−12^	6.11× 10^−12^	6.11 × 10^−12^	6.11 × 10^−12^	Optimized by fitting
Frac	0.282	0.844	0.296	0.628	Optimized by fitting
k_pre_d_ (min^−1^)	0.489	0.155	1.38 × 10^−5^	5.75 × 10^−10^	Optimized by fitting
k_pre_j_ (min^−1^)	1.73 × 10^−3^	2.73 × 10^−3^	1.90 × 10^−5^	1.90 × 10^−5^	Optimized by fitting

**Table 3:** Disposition parameters for loratadine derived from 12 independent PK studies. The mean values were used as input for simulation of the disposition of the drug.

Study	K_a_ (h^−1^)	K_10_ (h^−1^)	K_12_ (h^−1^)	K_21_ (h^−1^)	V/F (L)	Reference
1	1.131	0.079	1.025	0.032	474	[21]
2	0.962	0.2975	0.392	0.325	1791	[22]
3	0.509	0.116	0.407	0.001	1418	[23]
4	0.756	0.102	0.648	0.005	1342	[24]
5	0.819	0.088	0.726	0.007	1531	[25]
6	1.149	0.061	1.035	0.042	1083	[26]
7	1.434	0.684	0.669	0.098	859	[27]
8	0.798	0.797	9.869 × 10^−6^	0.062	1342	[28]
9	0.726	0.728	9.515 × 10^−6^	0.061	1526	[29]
10	0.837	0.181	0.700	4.933 × 10^−6^	1081	[30]
11	0.511	0.084	0.434	0.002	1112	[31]
12	0.523	0.039	0.518	0.010	820	[32]
**Mean**	**0.846**	**0.271**	**0.546**	**0.054**	**1198**	

K_a_ stands for the absorption rate constant; K_10_, K_12_, and K_21_ represent the disposition parameters with respect to elimination and distribution of the drug from the central to the peripheral compartment and vice versa. ‘V/F’ represents the apparent volume of distribution after an extravascular dose. The mean values were used as input for the modeling and simulation (M&S) in Phoenix WinNonlin^®^. The equations that were used to describe the mass transport analysis (MTA) and the distribution and clearance of the drug are provided in the supplementary materials (S2).

## Supplementary Materials

The following are available online at https://doi.org/10.5281/zenodo.3833818.

## Abbreviations

ASD	artificial stomach-duodenum model
AUC	area under the curve
BCS	biopharmaceutics classification system
C_max_	maximal concentration
CO_2_	carbon dioxide
CYP	Cytochrome P450
DS	degree of supersaturation
EMA	European Medicines Agency
FaSSGF	fasted state simulated gastric fluids
FaSSIF-v1	fasted state simulated intestinal fluids version 1
GI	gastrointestinal
GIS	gastrointestinal simulator
GIS_Duodenum_	duodenal chamber of the GIS
GIS_Jejunum_	jejunal chamber of the GIS
GIS_Stomach_	gastric chamber of the GIS
HPLC	high-performance liquid chromatography
HRM	high-resolution manometry
IR	immediate-release
k_sec_d_	secretion rate constant constant occuring in the duodenal chamber of the GIS
k_sec_s_	secretion rate constant constant occuring in the gastric chamber of the GIS
M&S	modeling and simulation
MTA	mass transport analysis
PBBM	physiologically-based biopharmaceutics modeling
PBPK	physiologically-based pharmacokinetics modeling
PDMA	Pharmaceuticals and Medical Devices Agency
PK	pharmacokinetics
PPI	proton-pump inhibitor
RLD	reference listed drug
t_1/2,G_	gastric half-life of emptying
TFA	trifluoroacetic acid
U.S. FDA	United States Food and Drug Administration
USP	United States Pharmacopoeia
V_d_	volume in the duodenal chamber of the GIS
V_j_	volume in the jejunal chamber of the GIS
V_s_	volume in the gastric chamber of the GIS
Z_D	duodenal dissolution coefficient, considering the pH-dependent dissolution and solubility of loratadine
Z_J	jejunal dissolution coefficient, considering the pH-dependent dissolution and solubility of loratadine
Z_S	gastric dissolution coefficient, considering the pH-dependent dissolution and solubility of loratadine
