# Erratum: Hens, B.; et al. Application of the Gastrointestinal Simulator (GIS) Coupled with In Silico Modeling to Measure the Impact of Coca-Cola^®^ on the Luminal and Systemic Behavior of Loratadine (BCS Class 2b). *Pharmaceutics*, 2020, *12*, 566

**DOI:** 10.3390/pharmaceutics12121137

**Published:** 2020-11-25

**Authors:** Bart Hens, Marival Bermejo, Patrick Augustijns, Rodrigo Cristofoletti, Gregory E. Amidon, Gordon L. Amidon

**Affiliations:** 1 Department of Pharmaceutical Sciences, College of Pharmacy, University of Michigan, Ann Arbor, MI 48109-1065, USA; bart.hens@kuleuven.be (B.H.); mbermejo@umh.es (M.B.); geamidon@umich.edu (G.E.A.); 2 Department of Pharmaceutical and Pharmacological Sciences, Faculty of Pharmaceutical Sciences, KU Leuven, Herestraat 49, 3000 Leuven, Belgium; patrick.augustijns@kuleuven.be; 3 Department Engineering Pharmacy Section, Miguel Hernandez University, San Juan de Alicante, 03550 Alicante, Spain; 4 Center for Pharmacometrics and Systems Pharmacology, Department of Pharmaceutics, College of Pharmacy, University of Florida, Orlando, FL 32827, USA; rcristofoletti@cop.ufl.edu

The authors make the following correction to this paper after the final publication of the work [1]: The authors acknowledge that Dr. Patrick Augustijns should have been included as one of the co-authors because of the conceptualization of the work with respect to the subject ‘real-life dosing conditions’.

The Author Contributions section has been updated as follows: B.H. and M.B. designed the experiments. Experimental work and ADMET Predictor simulations were performed by B.H. and M.B. performed the modeling experiments. P.A. conceptualized the research subject. G.L.A and G.E.A. supervised and co-financed the project. R.C. reviewed and edited the manuscript. All authors have read and agreed to the published version of the manuscript.
